# Comparison of G8 and ISAR Screening Results in Geriatric Urology

**DOI:** 10.3390/medicines8080040

**Published:** 2021-07-22

**Authors:** Jobar Bouzan, Boris Stoilkov, Spyridon Nellas, Marcus Horstmann

**Affiliations:** 1 Department of Urology, Helios Hospital St. Josefshospital, Krefeld Uerdingen, Kurfürstenstr. 69, 47829 Krefeld, Germany; jobar.bouzan@malteser.org (J.B.); boris.stoilkov@malteser.org (B.S.); spyridon.nellas@malteser.org (S.N.); 2 Department of Urology, University of Duisburg-Essen, Hufelandstr. 55, 45147 Essen, Germany

**Keywords:** geriatric screening, geriatric assessment G8 score, ISAR score, geriatric urology

## Abstract

Background: The G8 and ISAR scores are two different screening tools for geriatric risk factors and frailty. The aim of this study was to compare the G8 and ISAR screening results in a cohort of urogeriatric patients to help clinicians to better understand and choose between the two tests. Methods: We retrospectively evaluated 100 patients at the age of 75 and above who were treated for different urological conditions. All routinely and prospectively underwent G8 and ISAR screening tests. A G8 score ≤ 14 and an ISAR score > 2 were considered positive. The results for the two tests were compared and correlated to clinical data. Results: The mean age of the patients was 83 y (min. 75–max. 101); 78 of the patients were male, while 22 were female; 58 of the patients were G8-positive, while 42 were G8-negative; and 24 were ISAR-positive, while 76 ISAR were negative. All the ISAR-positive patients were also G8-positive. There was a significant negative correlation between the G8 and ISAR scores (r = −0.77, *p* < 0.001). Both tests correlated significantly with the Charlson comorbidity index, length of stay, number of coded diagnosis, and Braden score (*p* < 0.05). Conclusion: Both tests are significantly correlated with each other and to clinical data related to geriatric frailty. However, the G8 score has a much higher rate of positive tests, which limits its use in daily routine, and the ISAR score is therefore preferable. For “fit” geriatric patients, however, a negative G8 score can be of great use as a confirmatory test for further decision making.

## 1. Introduction

Because of an increased life expectancy and aging in recent years with high birth rates, geriatric aspects have become more and more important in the daily routine of urologists [1]. In addition, most urological malignancies, such as prostate, bladder, and renal cancer, as well as urinary retention or urinary tract infections and their subsequent medical, surgical, and/or radio-oncologic treatments, affect old and very old patients [2,3]. For optimal treatment, their geriatric situation should be assessed and included in medical decision making [4,5]. Currently, this is best evaluated by a fully comprehensive geriatric assessment (CGA) [4]. A CGA, however, is time- and resource-consuming and cannot be performed on every patient. As a solution, short geriatric assessments were developed as screening tools [6,7]. Among them are the G8 score and the ISAR score, which are both recommended as geriatric screening tests and are routinely performed in our department. Both tests were designed to measure geriatric frailty or geriatric risk factors for frailty. Frailty is herein defined as an increased vulnerability resulting from an age-related decline in reserve and function across multipole physiologic systems, such that the ability to cope with everyday or acute stressors is compromised [8]. Frailty carries an increased risk for poor health outcomes, including falls, incident disability, hospitalization, and mortality [8].

In the present study, we compared the results of the two screening tests and clinical data in our clinical routine of everyday patients in a German hospital of basic and regular care. The reason for this was that little is known regarding the differences between test results and their applicability in daily routine. The G8 score was chosen in the present study because it is intensively discussed in geriatric oncology [9], because it is recommended by urogeriatric guidelines [10] and because it has also shown its value in the screening of patients with benign conditions [11]. Cavusoglu et al. recently described a strong concordance between their G8 screening results and a complete geriatric assessment (CGA) in 200 nononcologic patients and therefore also proposed its use in these patients [11]. The ISAR score was chosen because it became a mandatory screening tool for all hospitalized patients of 75 years and above in our region of North Rhine Westphalia, Germany in 2015 [12]. Even though it was primarily designed for geriatric emergency patients [13], it has also been evaluated in oncological patients [14,15] and in nonemergency patients [16]. In our region, it is therefore considered as a standard routine test for all patients. The aim of the study was to help clinicians to better choose between the two screening tests and increase their understanding of them. 

## 2. Methods

**Figure A1:**
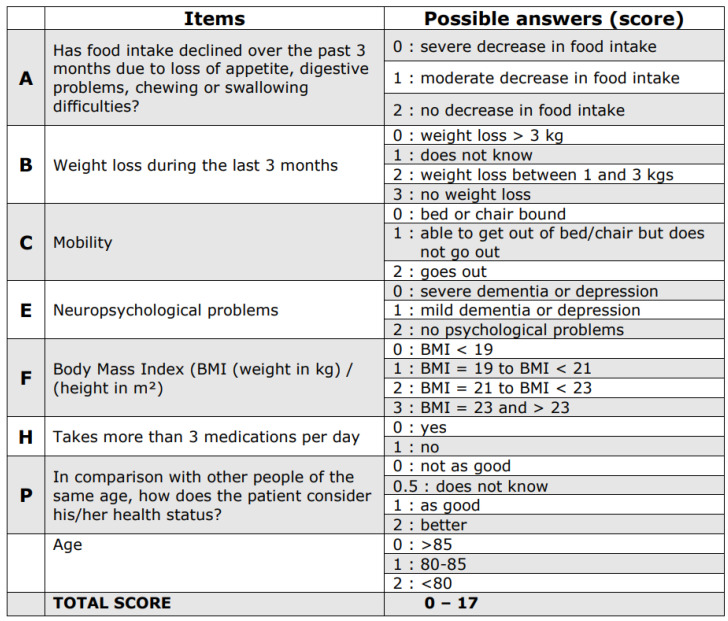
G8 Score.

One hundred patients aged 75 years and above, who were treated for different urological problems at the St. Josefshospital in Krefeld Uerdingen, were retrospectively included in the study. All the patients were hospitalized between 2018 and 2020. All underwent a prospective G8 and an ISAR screening at admission to the hospital. The G8 score is a questionnaire of 8 items [17,18]. Three items deal with nutritional items, while the others cover the area of mobility, neuropsychological restrictions, polypharmacy, age, and a comparison of the subjective health status with peers. It can be filled out by a medical person together with the patient in 5–8 min. The patients who reach more than 14 points of the maximum of 17 pts. are considered as “fit” geriatrics. The others have a positive test and are considered at risk of geriatric frailty. This was validated by Bellera et al. in oncologic patients with a high sensitivity at the cost of a lower specificity [17]. The test is depicted in Figure A1 in Appendix A. 

The ISAR (identification of patients at risk) score [19] is an assessment of 6 yes/no items that cover the area of need of help (2 items), prior hospitalizations, sensory restrictions (vision), cognitive impairment, and multimorbidity (polypharmacy). It can be filled out by medical persons in less than 5 min. A score higher than 2 of a maximum of 6 points is considered positive for geriatric risk factors or frailty. Initially, it was introduced and most intensively evaluated as a tool for emergency units [20]. However, it has recently become, in our region, a general tool for all hospitalized patients [12,16]. The ISAR questionnaire is depicted in Table A1 in Appendix B. 

Both the G8 and the ISAR tests were prospectively performed by residents of urology (J.B., B.S., S.N.) at admission. A G8 score ≤ 14 points and/or an ISAR score > 2 points were considered positive for risk of geriatric frailty. Patients with a G8 score > 14 or a ISAR score < 2 pts. were classified as “fit” geriatric patients. The patients’ characteristics were gathered retrospectively from their charts. These data included the age, gender, reasons for admission, treatment, length of stay, number of coded diagnosis, Charlson comorbidity index [21], and Braden score [22], which is an assessment for the risk of decubitus. Descriptive statistics were performed by Excel, Microsoft, 2016, Version 15.34. Comparisons were performed by Wilcoxon signed rank tests for dependent test values and the Kruskal Wallis tests for comparisons of more than three independent test values. The correlations were calculated by Spearman. All the statistics were performed using the SPSS software, IBM, Version 27. The study was performed according to the ethical standards of the Medical Council of North Rhine Westphalia, Germany. It required no ethical approval due its retrospective nature, with an evaluation of routine data (248/2020).

## 3. Results

The mean age of the patients was 83 years (min. 75y–max. 101y); 78 of the patients were male, and 22 were female; 24 of the patients were emergency patients, and 76 were regular admissions; 56 of the patients were treated due to urologic malignancies, and 44 were treated due to benign urologic conditions; and 73 patients underwent surgical procedures, while 27 underwent conservative treatments. The patients’ characteristics are listed in Table 1.

Of the patients, 58 were G8-positive, and 42 patients were G8-negative; and 24 of the patients were ISAR-positive, with 76 ISAR-negative. All the 24 ISAR-positive patients were G8-positive; and 34 of the patients were G8-positive, with ISAR-negative. There was a significant negative correlation between G8 and ISAR scores, r = −0.773, *p* < 0. 0001 (Table 2).

The highest G8 scores (mean 15.6 pts.) and the lowest ISAR scores (mean 0.4 pts.) were found in patients with a negative G8 score and a negative ISAR score (n = 42). The lowest G8 scores (mean 9.0 pts.) and highest ISAR scores (mean 4.0 pts.) were found in patients with a positive G8 score and a positive ISAR score (n = 24). These differences were significant (*p* < 0.001). For further details see Table 3.

Because of its test design, the G8 score becomes positive with a decreasing score. The three most frequent items, the score points of which were lost in our study, were: 1. age (>80 years), 2. H: polypharmacy (>3 drugs), and 3. B: weight loss. Of the patients, 75 lost 1–2 points for an age over 80 years; 67 of the patients lost 1 point in item H due to a polypharmacy of more than 3 drugs; and 49 of the patients lost 3 points or less in item B due to a weight loss of 1 kg to > 3 kg/3 months.

In contrast to the G8 score, the ISAR score becomes positive with an increasing score from three to a maximum of six. The three items of the ISAR score, the score points of which were most often gained in our study, were: 1. polypharmacy (>6 drugs) (n = 49), 2. hospitalization during the last 6 months (n = 32), and 3. constant need of help (n = 31). The results of the two tests and their combinations were compared with the Charlson comorbidity index, length of hospital stay, number of coded diagnosis in our DRG system, and Braden score. All the parameters revealed a significant correlation to both the G8 and the ISAR score results (Table 2). The lowest Charlson comorbidity index, the shortest length of stay, the lowest number of coded diagnosis, and the highest Braden score were found in patients with a negative G8 score (n = 42 pat.). In contrast, the highest Charlson index, the longest length of stay, the highest number of coded diagnosis, and the lowest Braden score were found in patients with a positive ISAR score (n = 24 pat.). For further details, see Table 3.

## 4. Discussion

In urology, the clinical need for geriatric assessments is high [1]. The reasons for this are the aging of our society and an age-related increase of most urological medical conditions. Both the G8 score and the ISAR score are recommended as screening tests for geriatric risk factors [12,23,24]. Until now, reports and comparisons between them in the literature are rare but important in evaluating their applicability in clinical routine and making a choice between them. Only two reports were found in the literature in which both tests were applied [14,15]. In 139 patients above 70 years, Souwer et al. evaluated the G8 score and the ISAR—HP score for the prediction of adverse surgical outcomes in colorectal surgery [14]. The ISAR—HP score was positive in 32 patients, and the G8 score was positive in 68 patients. The authors reported that ISAR—HP-positive patients had a significantly higher risk of a 30-day complication. Schulkes et al. evaluated the G8 and the ISAR HP score in 142 patients with lung cancer. Potentially frail patients with positive scores had a significantly greater risk of 1-year mortality. Patients with a positive G8 and ISAR HP score had more geriatric impairments than patients with only a positive G8 score [15].

In the present study, we compared the results of the G8 and the ISAR score in an unselected group of urogeriatric patients of our department and compared both to clinical data. The reason for this was that both tests are already, as mentioned above, used for general screening and not only in cancer patients in the case of the G8 score and in emergency patients in case of the ISAR score. The most important difference between the two tests was, in our study, that the number of positive G8 scores was much higher than that of the ISAR scores. These findings are similar to those of Souwer et al. [14]. While 58 patients had a positive G8 score, only 26 had a positive ISAR score in our study. This is in line with the literature, in which a high sensitivity and low specificity of the G8 score are described [14,18,25].

The main reason for the high number of positive G8 scores can be explained by its test design, which favors sensitivity over specificity. Already at a cut off of 14 points or less, the G8 score turns positive. If only 3 points are not achieved, the test becomes positive. Furthermore, our evaluation shows that the score points are easily lost in several items of the G8 score. For example, 78 of the patients lost one or two points due to their age of 80 years or higher, and 67 of the patients lost a score point for polypharmacy due to a medication of more than 3 drugs. In contrast, the threshold for polypharmacy using the ISAR score is above 6, and age is not an item in it. Together with the fact that several items of the G8 score additionally rely on subjective estimations, these examples explain the higher rate of positive G8 scores in comparison to ISAR-positive scores. 

Even though highly different in their rate of positive and negative test results, the two tests had a significant negative correlation with each other. They also revealed a significant and equal correlation to important clinical parameters: length of stay, Charlson comorbidity index, number of coded diagnosis, and the Braden score (Table 2). The latter factors are all known to be related to geriatric risk factors and frailties [12]. Together with the fact that none of the patients who were ISAR-positive were G8-negative, these correlation data give us evidence that both tests equally measure geriatric risk factors and frailty in our population. Similar to the results of Schulkes et al. in pulmonary cancer patients, patients with a positive G8 and ISAR score were considered to be clinically the frailest [15]. This interpretation, however, has to be handled with care, because the results of the two tests were not verified by a comprehensive geriatric assessment, which is the current golden standard for geriatric frailties [4,5]. Because, until now, comprehensive geriatric assessments were not part of our clinical hospital routine, they were unfortunately not available in the present study. This is a major limitation of our study. Another limitation is that the study design and the heterogeneity of the study population do not allow for a predictive outcome evaluation of both tests.

Nevertheless, the study helps to better apply both tests in daily routine. A negative G8 score seems to us most suitable as a confirmatory test for “fit” geriatric patients, because patients with a negative G8 score were those with the lowest geriatric risk factors and were, in our cohort, the fittest. A negative G8 score can be ideally used in decision making for the treatment of urogeriatric patients with malignancies, prior to major interventions, such as curative surgery, radiation therapy, or palliative chemotherapy. This is in line with the current guidelines of the international society of geriatric oncology (SIOG) and supports their recommendation to use the G8 score as a routine test for prostate cancer patients older than 70 years [10,23]. Because of the high positive rate, the G8 score, however, is in our eyes less suitable for the routine testing of mixed patient groups, at least in our study. In this setting, the ISAR screening is advantageous, because it has a higher specificity. Still, it proved to be a robust test, which detected the most critical patients in our cohort. 

To conclude our study, the two tests were significantly correlated with each other and with important clinical data related to geriatric frailty. Because the G8 score has a much higher rate of positive tests, its daily routine use is limited, and the ISAR score is preferable. For “fit” geriatric patients, however, a negative G8 score can be of great use as a confirmatory test for further decision making.

## Figures and Tables

**Table 1:** Patients’ characteristics and results. Abbreviations: M = male, f = female, y = years, d = days.

	All
Patients (m/f), (n = pat.)	100 (78/22)
Age, (y, min–max)	83.25 (75–101)
malignancy, (n = pat.)	56
benign disease, (n = pat.)	44
*prostate cancer*	*24*
*urothelial cancer*	*28*
*renal cancer*	*4*
*urolithiasis*	*4*
*benign subvesical obstruction*	*21*
*urinary tract infections*	*9*
*upper urinary tract retention*	*4*
*hematuria*	*6*
Results	
G 8 score (mean (min–max))	12.9 (4–17)
ISAR score (mean (min–max))	1.6 (0–6)
Charlson comorbidity score (mean (min–max))	3.1 (0–7)
length of hospital stay, (n = d), (mean (min–max))	7.9 (1–24)
number of coded diagnosis, (mean (min–max))	9.5, (2–32)
Braden score, (mean (min–max))	18.25 (9–23)

**Table 2:** Correlation (Spearman) between G8 and ISAR score results and age, Charlson comorbidity index, length of stay (d), number of coded diagnosis, and Braden score. Abbreviations: d = days.

		G8 Score	ISAR Score	Age	Charlson Comorbidity Index	Length of Stay (d)	Number of Coded Diagnosis	Braden Score
G8 score	Correlation coefficient, Spearman	1	−0.773	−0.599	−0.368	−0.337	−0.418	0.278
	*p*-value		0.000	0.000	0.000	0.001	0.000	0.005
ISAR score	Correlation coefficient, Spearman	−0.773	1	0.393	0.341	0.218	0.487	−0.264
	*p*-value	0.000		0.000	0.001	0.029	0.000	0.008

**Table 3:** Mean values of the G8 score, ISAR score, and clinical data of different patient groups. Groups were built according to the score results (positive/negative screening tests).

	1. Pat. with Negative G8 Scores (n = 42 pat.)	2. Pat. with Negative ISAR Scores (n = 76 pat.)	3. Pat. with Positive G8 and Negative ISAR Scores (n = 34 pat.)	4. Pat. with Positive G8 Scores (n = 58 pat.)	5. Pat. with Positive ISAR Scores (n = 24 pat.)	*p*-Values
G8 score (mean values)	15.6	14.1	12.4	11.0	9.0	*p* < 0.001
ISAR score (mean values)	0.4	0.8	1.3	2.4	4.0	*p* < 0.001
Charlson comorbidity Index (mean values)	2.40	2.94	3.61	3.65	3.70	*p* < 0.003
Length of stay, days (mean values)	6.14	7.38	8.91	9.20	9.62	*p* < 0.001
Number of coded diagnosis (mean values)	6.73	8.23	10.08	11.46	13.41	*p* < 0.001
Braden score (mean values)	19.42	18.88	18.20	17.39	16.25	*p* < 0.001

## Data Availability

The data used in this study can be made available upon reasonable request from the corresponding author. The following are available: SPSS, licensed by University of Essen, Excel 02984-001-000001, and Word 02985-010-000001.

## Appendix B

**Table A1:** ISAR Score.

	Yes	No
Before the illness that brought you to the hospital, did you need someone to help you on a regular basis?	1	0
Since the illness or injury that brought you to the hospital, have you needed more help than usually before?	1	0
Have you been hospitalized for more for one or more nights during the past 6 months?	1	0
In general, is your sight (with glasses) good?	0	1
In general, do you have serious problems with your memory?	1	0
Do you take more than six different medications every day?	1	0
